# The Limitation of HLA Diversity as a Risk Factor for Pediatric-Onset Autoimmune Rheumatic Disease

**DOI:** 10.3390/jcm14030916

**Published:** 2025-01-30

**Authors:** Ioannis Kalampokis, Craig S. Wong, Jihyun Ma, Lynette M. Smith, Barbara J. Masten, Devon Chabot-Richards, David S. Pisetsky

**Affiliations:** 1 University of Nebraska Medical Center, Omaha, NE 68198, USA; jihyun.ma@unmc.edu (J.M.); lmsmith@unmc.edu (L.M.S.); 2 University of New Mexico, Albuquerque, NM 87106, USA; cwong@salud.unm.edu (C.S.W.); bmasten@salud.unm.edu (B.J.M.); dchabot-richards@salud.unm.edu (D.C.-R.); 3 Tricore Reference Laboratories, Albuquerque, NM 87102, USA; 4 Duke University Medical Center, Durham, NC 27710, USA; david.pisetsky@duke.edu; 5 Durham Veterans Administration Medical Center, Durham, NC 27705, USA

**Keywords:** autoimmunity, HLA, homozygosity, genetic diversity, immunogenetics

## Abstract

**Background:** HLA homozygosity of specific alleles at a single locus is associated with increased risk for autoimmunity and/or more severe clinical phenotypes. However, the contribution of the overall limitation of HLA diversity across multiple loci to autoimmunity risk remains to be determined. **Methods:** We conducted a proof-of-concept case–control study of 413 individuals (279 cases with pediatric-onset autoimmune rheumatic diseases, 134 matched controls) examining the “Limitation of HLA Diversity” (LoHLAD) across multiple loci as an allele-independent risk factor for autoimmunity. We examined the association of LoHLAD with pediatric-onset autoimmune rheumatic diseases at five HLA loci (A, B, DQB1, DRB1, DRB3/4/5). LoHLAD was defined as (1) homozygosity at any of the examined loci, and/or (2) the presence of a single allele or the complete lack of an allele at the HLA-DRB3/4/5 locus. **Results:** The frequency of LoHLAD at any locus was significantly higher in cases compared to controls (65.95% vs. 30.60%, OR 4.39 [2.82–6.84], *p* < 0.0001). Higher frequencies of LoHLAD in cases compared to controls were observed at both class I (19.35% vs. 10.45%, OR 2.06 [1.10–3.86], *p* = 0.031) and class II (54.48% vs. 20.15%, OR 4.74 [2.92–7.69], *p* < 0.0001) loci. Specifically, significant differences between cases and controls were observed at the B (OR 8.63 [1.14–65.55], *p* = 0.016), DQB1 (OR 3.34 [1.27–8.78], *p* = 0.016), and DRB3/4/5 (OR 4.64 [2.77–7.75], *p* < 0.0001) loci. Multiple logistic regression models confirmed the ability of LoHLAD to positively predict autoimmunity. **Conclusions:** LoHLAD is a significant allele-independent risk factor for pediatric-onset autoimmune rheumatic disease.

## 1. Introduction

Human leukocyte antigen (HLA) genes encode for cell-surface glycoproteins that present antigenic peptides to T cell receptors and play key roles in the induction of adaptive immune responses [1]. HLA genes are located at the major histocompatibility complex (MHC) locus on human chromosome 6p21.3. The MHC locus is among the most polymorphic genetic loci in the human genome [2]. The extreme polymorphism of HLA genes combined with the codominant expression of chromosomal alleles constitutes the basis for the extensive diversity in antigen presentation. This diversity enables the presentation of a broad range of epitopes to T cells, thus potentially maximizing the repertoire of immune responses to non-self antigenic challenges.

Maximum diversity in antigen presentation is attained through HLA heterozygosity. An antigen-presenting cell (APC) from a heterozygous individual at each of the six classical HLA class I or II loci could theoretically present over 10^12^ different peptides [3]. Studies suggest that MHC heterozygosity provides an advantage against infectious diseases both in mice [4] and humans [5]. This heterozygote advantage is one of the main mechanisms postulated to favor the maintenance of HLA diversity in a population over time [6]. Furthermore, in animal models, restriction of the MHC–peptide repertoire has been shown to predispose to autoimmunity. For example, genetically engineered mice with an MHC class II–peptide repertoire reduced to a single complex demonstrate various autoimmune reactions [7].

While the MHC locus is the single genomic region associated with the greatest number of human diseases [8,9], including autoimmune diseases, the mechanisms by which HLA genes affect the risk of autoimmunity have not been fully elucidated. Historically, the contribution of HLA genes to the development of autoimmunity has been examined predominantly from the standpoint of specific HLA allele and/or haplotype associations with particular disease phenotypes [10,11]. Furthermore, the presence of HLA homozygosity of specific high-risk alleles such as HLA-B27 [12,13,14,15,16,17,18,19,20,21] has been postulated to worsen the clinical manifestations of autoimmunity by means of a “gene dosage effect”. We propose that the documented effect of HLA homozygosity in autoimmunity may be alternatively explained by the limitation of HLA diversity and the resulting constraint of the heterozygote advantage.

To our knowledge, the potential role of the limitation of HLA diversity in human autoimmunity has not been characterized. Thus, we tested the hypothesis that the limitation of HLA diversity across multiple loci is a distinct risk factor for autoimmune disease independent of allele specificity. We conducted a proof-of-concept case–control study examining the limitation of HLA diversity across multiple loci (A, B, DQB1, DRB1, DRB3/4/5) as an allele-independent risk factor for pediatric-onset autoimmune rheumatic disease. In this context, we introduced the novel concept of “Limitation of HLA Diversity” (LoHLAD) which, for the purpose of our study, was defined as (1) homozygosity at any of the 5 examined loci, and/or (2) the presence of either a single allele or the complete lack of an allele at the HLA-DRB3/4/5 locus. As the results of our study indicate, LoHLAD is a significant risk factor for pediatric-onset autoimmune rheumatic disease.

## 2. Methods

### 2.1. Study Approval

This research study involves human participants and was approved by the institutional review board of the University of New Mexico (20-054). The study was performed according to the medical research ethical principles of the World Medical Association Declaration of Helsinki and was compliant with Health Insurance Portability and Accountability Act (HIPAA) regulations. Consent was waived because only de-identified data were used.

### 2.2. Study Population

Retrospective review of electronic medical records of all patients evaluated at the University of New Mexico pediatric rheumatology clinic between November 2016 and January 2021 was performed. HLA typing was obtained during laboratory evaluation of children referred to the pediatric rheumatology clinic. Inclusion criteria consisted of (1) availability of HLA data for all 5 HLA loci (see “HLA typing” below), and (2) diagnosis of pediatric-onset (prior to 16 years of age) autoimmune rheumatic disease (“cases”) or non-autoimmune common musculoskeletal conditions (“controls”). Cases consisted of patients with juvenile idiopathic arthritis (JIA), systemic lupus erythematosus (SLE), chronic idiopathic uveitis, mixed connective tissue disease (MCTD), undifferentiated connective tissue disease (UCTD), localized scleroderma (LoS), vasculitis, juvenile dermatomyositis (JDM), Sjögren’s syndrome (SS), and systemic sclerosis (SSc). Controls did not satisfy the diagnostic criteria for any autoimmune rheumatic disease, and consisted of patients with benign hypermobility, orthopedic conditions, and pain amplification syndromes. Patients with isolated (primary) Raynaud’s phenomenon or autoinflammatory conditions such as periodic fever syndromes (Familial Mediterranean Fever; Periodic Fever with Aphthous stomatitis, Pharyngitis and Adenitis syndrome; Tumor Necrosis Factor Receptor Associated Periodic Syndrome; Mevalonate Kinase Deficiency) were excluded because our study focus is autoimmune rheumatic disease.

### 2.3. Diagnostic Criteria

Cases with JIA satisfied the International League of Associations for Rheumatology (ILAR) criteria [22]. Cases with SLE satisfied the 2019 European League Against Rheumatism/American College of Rheumatology (EULAR/ACR) classification criteria [23]. Cases with chronic idiopathic uveitis were diagnosed after ruling out all known causes of uveitis and following evaluation by a pediatric ophthalmologist. Cases with MCTD/UCTD had high-titer (≥1:1280) positive anti-nuclear antibody (ANA) testing, negative anti-double stranded DNA testing, Raynaud’s phenomenon plus at least one of the following: synovitis, myositis, sclerodactyly, cytopenia, or interstitial lung disease. Testing for anti-U1 ribonucleoprotein (anti-U1RNP) antibody was positive in MCTD and negative in UCTD. LoS was confirmed by skin biopsy in all cases. Cases with ANCA-positive and ANCA-negative vasculitis had evidence of vasculitis on angiography and/or tissue biopsy. Cases with IgA vasculitis satisfied the EULAR/PRINTO/PRES (European League Against Rheumatism/Paediatric Rheumatology International Trials Organisation/Paediatric Rheumatology European Society) criteria [24]. The single case of Behçet’s disease satisfied the pediatric Behçet’s disease criteria [25]. Cases with JDM satisfied the EULAR/ACR criteria [26]. Cases with SS satisfied the ACR/EULAR criteria [27]. Cases with SSc satisfied the PRES/ACR/EULAR criteria [28].

### 2.4. HLA Typing

Genomic DNA was extracted from whole blood samples using the Qiagen EZ1 Blood Kit and the EZ1 Advanced or EZ1 Advanced XL automated purification instrument (Qiagen, Germantown, MD, USA). HLA-A, HLA-B, HLA-DRB1, HLA-DRB3/4/5, and HLA-DQB1 genotyping was performed using a microarray bead-based reverse sequence specific oligonucleotide (SSO) typing methodology (LABType^TM^ [rSSO], One Lambda, A Thermal Fisher Scientific Brand, West Hills, CA, USA) and the LABScan3D™ instrumentation with Luminex^®^ xPONENT^®^ software version 4.2 for data acquisition (One Lambda, A Thermal Fisher Scientific Brand, West Hills, CA, USA). Genotype data was analyzed using HLA Fusion^TM^ software version 4.4 (One Lambda, A Thermal Fisher Scientific Brand, West Hills, CA, USA).

### 2.5. Statistical Analysis

We examined 5 HLA loci (A, B, DQB1, DRB1, DRB3/4/5). The term “Limitation of HLA Diversity” (LoHLAD) was defined as (1) homozygosity at any of the 5 examined loci, and/or (2) the presence of a single allele or the complete lack of an allele at the HLA-DRB3/4/5 locus. The HLA-DRB3/4/5 locus is in linkage disequilibrium with the HLA-DRB1 locus; DRB1*01, DRB1*08 and DRB1*10 are associated with the lack of an allele at the HLA-DRB3/4/5 locus [29]. Descriptive statistics for demographics were summarized as means, standard deviations, standard error of means, medians, inter-quartile ranges, odds ratios (OR) with 95% confidence intervals (CI), counts, and percentages. The distributions of patient characteristics between controls and cases, and the association of LoHLAD or specific alleles with pediatric-onset autoimmune rheumatic diseases were examined using Fisher’s exact test. Risk estimates are presented as OR. The Cochran–Armitage trend test was used to determine the cumulative effect of LoHLAD by comparing the distribution of the number of loci with LoHLAD (“hits”) between cases and controls. The OR of the number of “hits” were calculated in conjunction with cumulative outcomes to demonstrate quantifiable effects of LoHLAD. Logistic regression modeling evaluated the ability of LoHLAD or alleles to predict autoimmune rheumatic disease. The concordance between observed and predicted outcomes (“c-statistic”) from univariate and multivariate logistic regression models was used to identify the best fitted models. A model with a c-statistic ranging from 0.70 to 0.80 has an adequate power of discrimination; a range of 0.80 to 0.90 is considered excellent [30]. *p* values were adjusted using the “False Discovery Rate” (FDR) correction for multiple comparisons [31]. Missing values were excluded from all analyses. A *p* value < 0.05 was considered significant. SAS version 9.4 (SAS Institute Inc., Cary, NC, USA) was used for all analyses.

## 3. Results

### 3.1. Demographics and Clinical Parameters

To identify patients with pediatric-onset autoimmune rheumatic diseases and controls, the electronic medical records of 516 individuals were reviewed. We excluded 103 individuals from the analysis: 81 were excluded due to lack of *HLA* typing data, and 22 were excluded due to their diagnosis of an autoinflammatory condition or primary Raynaud’s phenomenon (Supplementary Figure S1). A total of 413 individuals (279 cases and 134 controls) satisfied the inclusion criteria (see Methods and Patients). No statistically significant differences were noted between cases and controls regarding sex, race, or ethnicity (Table 1). We observed significantly higher frequency of positive family history of autoimmunity in cases compared to controls (Table 1; OR 2.00 [1.30–3.10]; *p* = 0.002). The age of cases (mean 12.7 years, median 13.1 years) was significantly lower than the age of controls (mean 13.5 years, median 14.6 years) (Table 1; *p* = 0.029). The distribution of specific pediatric-onset autoimmune rheumatic disease diagnoses in our cohort is presented in Supplementary Table S1.

### 3.2. LoHLAD in Cases vs. Controls

**Figure 1:**
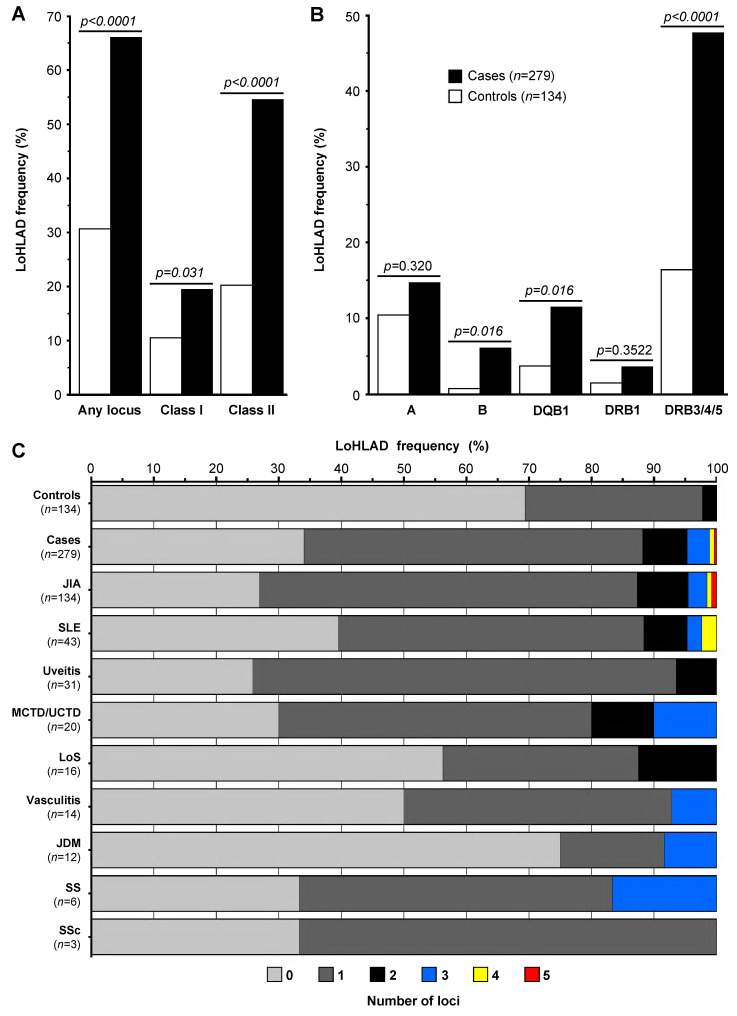
**Limitation of HLA Diversity (LoHLAD) frequency distributions**. (**A**,**B**) LoHLAD frequency distributions of cases (black bars, *n* = 279) and controls (white bars, *n* = 134) exhibited as grouped bar plots; LoHLAD frequencies displayed as the percentage of subjects that LoHLAD was present; *p* values calculated by Fisher’s exact test (corrected for multiple comparisons by False Discovery Rate). (**A**) Collective frequencies of LoHLAD at any of the 5 examined *HLA* loci (“Any locus”), *HLA-class I* loci, and *HLA-class II* loci in cases vs controls. (**B**) Frequencies of LoHLAD at individual loci (*HLA-A*, *HLA-B*, *HLA-DQB1*, *HLA-DRB1*, *HLA-DRB3/4/5*) in cases vs controls. (**C**) The cumulative effect of LoHLAD (number of loci with LoHLAD, range 0–5) is presented by displaying the frequency distributions (horizontal axis) of the number of loci with LoHLAD (colors) in controls, all cases, and specific pediatric-onset autoimmune disease diagnoses (vertical axis) as stacked bars. Bar colors correspond to the number of loci with LoHLAD (0: light gray, 1: dark gray, 2: black, 3: blue, 4: yellow, 5: red). JIA, Juvenile Idiopathic Arthritis; SLE, Systemic Lupus Erythematosus; MCTD, Mixed Connective Tissue Disease; UCTD, Undifferentiated Connective Tissue Disease; LoS, Localized Scleroderma; JDM, Juvenile Dermatomyositis; SS, Sjögren’s Syndrome; SSc, Systemic Sclerosis.

We assessed the effect of LoHLAD as a risk factor for pediatric-onset autoimmune rheumatic disease by comparing the frequency of LoHLAD between cases and controls as a binary event (present vs. non-present) (Table 2). First, we examined whether LoHLAD was present at any of the 5 examined loci (*HLA-A*, *HLA-B*, *HLA-DQB1*, *HLA- DRB1*, *HLA- DRB3/4/5*), and compared the frequency of LoHLAD between cases and controls. The frequency of LoHLAD at any locus was significantly higher in cases compared to controls (65.95% vs. 30.60%, OR 4.39 [2.82–6.84], *p* < 0.0001) (Figure 1A and Table 2). We then evaluated LoHLAD at the *HLA-class I* and *HLA-class II* level, and at the level of individual loci. Although the frequency of LoHLAD was significantly higher in cases compared to controls at both the *HLA-class I* and *HLA-class II* level, the difference was more prominent at *HLA-class II* (54.48% vs. 20.15%, OR 4.74 [2.92–7.69], *p* < 0.0001) compared to *HLA-class I* (19.35% vs. 10.45%, OR 2.06 [1.10–3.86], *p* = 0.031) loci (Figure 1A and Table 2). Examination of individual loci revealed significantly higher frequencies of LoHLAD in cases compared to controls at the *HLA-B* (6.09% vs. 0.75%; OR 8.63 [1.14–65.55], *p* = 0.016), *HLA-DQB1* (11.47% vs. 3.73%, OR 3.34 [1.27–8.78], *p* = 0.016), and *HLA-DRB3/4/5* (47.67% vs. 16.42%, OR 4.64 [2.77–7.75], *p* < 0.0001) loci (Figure 1B and Table 2). The association of LoHLAD with pediatric-onset autoimmune rheumatic disease at the *HLA-A* and *HLA-DRB1* loci was not statistically significant.

### 3.3. Cumulative Effect of LoHLAD (“Dose–Response Effect”)

We next examined the cumulative effect of LoHLAD as a risk factor for pediatric-onset autoimmune rheumatic disease by comparing the distribution of the number of “hits” (defined as the number of *HLA* loci with LoHLAD, range: 0–5) between cases and controls. The observed difference was significant (Cohran–Armitage trend test, *p* < 0.0001). Specifically, 0 “hits” were observed in 34.05% of cases vs. 69.40% of controls, 1 “hit” in 54.12% of cases vs. 28.36% of controls (OR 3.89 [2.47, 6.14], *p* < 0.0001), and 2 “hits” in 7.17% of cases vs. 2.24% of controls (OR 6.53 [1.88, 22.70], *p* = 0.0007) and 2 or more “hits” in 11.83% of cases vs. 2.23% of controls (OR 10.77 [3.19–36.33], *p* < 0.0001) (Table 3). Notably, 3 or more “hits” were observed exclusively in cases although at low frequency (4.66%). A graphic representation of the cumulative effect of LoHLAD as a risk factor for pediatric-onset autoimmune rheumatic disease is illustrated in Figure 1C.

### 3.4. LoHLAD in Specific Pediatric-Onset Autoimmune Rheumatic Diseases

After determining the differences in the frequency of LoHLAD between all cases and controls, we evaluated the association of LoHLAD with specific pediatric-onset autoimmune rheumatic disease diagnoses (Table 4) by comparing the frequency of LoHLAD between groups of cases with specific diagnoses and controls. Significantly higher frequencies of LoHLAD were observed in all specific disease diagnosis groups with 20 or more cases (JIA, SLE, chronic idiopathic uveitis, MCTD/UCTD) compared to controls. The strongest associations were observed in JIA (OR 6.18 [3.63–10.49], *p* < 0.0001) and chronic idiopathic uveitis (OR 6.52 [2.69–15.79], *p* < 0.0001). Furthermore, the distribution of the number of “hits” was significantly different between all specific disease diagnosis groups with 20 or more cases and controls (*p* < 0.0001, Supplementary Table S2). Within cases, we examined whether earlier symptom onset and shorter time to diagnosis is associated with LoHLAD. Cases with LoHLAD compared to cases without LoHLAD had significantly earlier symptom onset and shorter time to diagnosis (Supplementary Table S3).

### 3.5. LoHLAD-Based Models

Logistic regression models were constructed to evaluate LoHLAD as a positive predictor of pediatric-onset autoimmune rheumatic disease. The best fitted model included LoHLAD of 4 out of the 5 examined *HLA* loci (*HLA-A*, *HLA-B*, *HLA-DQB1*, *HLA-DRB3/4/5*) as separate binary variables (present vs. non-present). LoHLAD at the *HLA-DRB1* locus correlated significantly with LoHLAD at the *HLA-DQB1* locus and thus was excluded from the best fitted model to limit multicollinearity (Supplementary Table S4); the *HLA-DRB1* and *HLA-DQB1* loci are in linkage disequilibrium [32]. The c-statistic of the best fitted model was 0.711 for predicting any pediatric-onset autoimmune rheumatic disease, and 0.735 for predicting any of the 4 most common specific disease diagnoses in our cohort (JIA, SLE, chronic idiopathic uveitis, MCTD/UCTD) (Supplementary Table S5). The ability of the best fitted LoHLAD-based model to predict specific disease diagnoses was highest for JIA and chronic idiopathic uveitis, with c-statistic values of 0.749 and 0.769 respectively (Supplementary Table S5). Detailed evaluation of the best fitted LoHLAD-based model is presented in Supplementary Table S6.

### 3.6. Allele-Based Models

Logistic regression models were constructed to evaluate allele specificity as a positive predictor of pediatric-onset autoimmune rheumatic disease. Six alleles were identified as positive predictors for pediatric-onset autoimmune rheumatic disease: *HLA-A02*, *HLA-A24*, *HLA-B27*, HLA-DRB1*01, HLA-DRB1*08, and HLA-DQB1*04 (Supplementary Table S7. The best fitted allele-based model included 5 out of 6 alleles (*HLA-A02*, *HLA-A24*, *HLA-B27*, HLA-DRB1*01, HLA-DRB1*08). HLA-DQB1*04 strongly correlated with HLA-DRB1*08 (Supplementary Table S4); 73 out of 81 HLA-DRB1*08 positive individuals were HLA-DQB1*04 positive, and 73 out of 89 HLA-DQB1*04 positive individuals were HLA-DRB1*08 positive (*p* < 0.0001). Therefore, HLA-DQB1*04 was excluded from the best fitted model to limit multicollinearity (Supplementary Table S5). The linkage disequilibrium between HLA-DRB1*08 and HLA-DQB1*04 is well-established [33]. The c-statistic of the best fitted allele-based model was 0.698 for predicting any pediatric-onset autoimmune rheumatic disease, and 0.713 for predicting any of the 4 most common specific disease diagnoses in our cohort (JIA, SLE, chronic idiopathic uveitis, MCTD/UCTD) (Supplementary Table S5). The ability of the best fitted allele-based model to predict specific disease diagnoses was highest for JIA and chronic idiopathic uveitis, with c-statistic values of 0.753 and 0.780 respectively (Supplementary Table S5).

### 3.7. Composite Models

**Figure 2:**
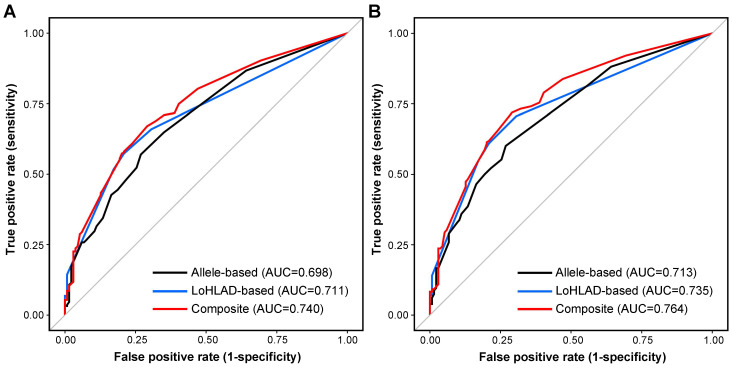
**Receiver Operating Characteristic (ROC) curves of best fitted models.** The ability of the best fitted multivariate logistic regression models in predicting the presence of (**A**) any pediatric-onset autoimmune rheumatic disease or (**B**) any of the 4 most common pediatric-onset autoimmune disease diagnoses (JIA, SLE, chronic idiopathic uveitis, MCTD/UCTD) in our cohort is presented as ROC curves. The Area Under the Curve (AUC) values are shown for allele-based models (black line), LoHLAD-based models (blue line), and composite models (red line). LoHLAD, Limitation of *HLA* Diversity; JIA, Juvenile Idiopathic Arthritis; SLE, Systemic Lupus Erythematosus; MCTD, Mixed Connective Tissue Disease; UCTD, Undifferentiated Connective Tissue Disease.

We constructed composite models by utilizing both LoHLAD and allele specificity as positive predictors of pediatric-onset autoimmune rheumatic disease (Supplementary Table S5). The best fitted composite model included 4 LoHLAD-based variables (*HLA-A*, *HLA-B*, *HLA-DBQ1*, *HLA-DRB3/4/5*) and 3 allele-based variables (*HLA-A02*, *HLA-A24*, *HLA-B27*). HLA-DRB1*01, HLA-DRB1*08 and HLA-DQB1*04 significantly correlated with LoHLAD at the *HLA-DRB3/4/5* locus and thus were excluded to limit multicollinearity (Supplementary Table S4). The c-statistic of the best fitted composite model was 0.740 for predicting any pediatric-onset autoimmune rheumatic disease, and 0.764 for predicting any of the 4 most common diagnoses in our cohort (JIA, SLE, chronic idiopathic uveitis, MCTD/UCTD) (Supplementary Table S5). The ability of the best fitted composite model to predict specific disease diagnoses was highest for JIA and chronic idiopathic uveitis, with c-statistic values of 0.796 and 0.822 respectively (Supplementary Table S5). The Receiver Operating Characteristic (ROC) curves of the best fitted LoHLAD-based, allele-based, and composite models are illustrated in Figure 2.

## 4. Discussion

In this proof-of-concept case–control study, we documented a 4-fold increased risk for pediatric-onset autoimmune rheumatic diseases associated with LoHLAD at any of the 5 examined loci. In addition, the risk for pediatric-onset autoimmune rheumatic disease increased as the number of loci with LoHLAD increased. Our findings indicate that LoHLAD is a significant genetic risk factor for the development of early-onset autoimmunity in humans. The assessment of the LoHLAD introduces a novel approach in the investigation of *HLA*-related genetic determinants of autoimmunity, extending beyond the well-established role of specific *HLA* alleles and haplotypes in autoimmune disease immunopathogenesis. The identification of LoHLAD as a risk factor for autoimmune rheumatic disease highlights the potentially beneficial role of the “*heterozygote advantage*” [6] outside of the realm of infectious diseases [4,5]. We theorize that LoHLAD increases the risk for autoimmunity by limiting the diversity of the MHC–peptide repertoire and thus restricting antigen-specific immune responses; in animal models, restriction of the MHC–peptide repertoire has been shown to predispose to autoimmunity [7]. The risk effect of LoHLAD on autoimmunity is further amplified in individuals with high-risk *HLA* alleles. Our theory proposes that the combination of high-risk *HLA* alleles and a restricted antigen presentation repertoire through LoHLAD results in the potential enrichment of adaptive immune responses for autoreactive T cells.

The detrimental effects of *HLA* homozygosity have been described in a variety of autoimmune diseases. For example, *HLA-B27* homozygosity is associated with increased phenotypic severity in ankylosing spondylitis [12,13,14,15,16,18,19]. The effects of *HLA* homozygosity in autoimmunity are not limited to *HLA-B27*. Patients homozygous for the HLA-Cw*06 allele have increased risk for developing psoriasis compared to heterozygotes [34]. HLA-DRB1*04 homozygosity is associated with severe extra-articular disease in rheumatoid arthritis (RA) [35,36,37]. Runs of homozygosity (ROH) at the human MHC locus are associated with RA [38]. *HLA-DQ2.5* heterodimer (HLA-DQA1*05-DQB1*02) homozygosity increases the risk for celiac disease (CD) [39] and enteropathy-associated T cell lymphoma [40]. Children homozygous for the haplotype *DR3-DQ2* are at high risk for early-onset CD [41]. The effects of *HLA* homozygosity have also been documented in immune thrombocytopenia [42], autoimmune liver disease [43,44,45,46], pemphigus vulgaris [47,48], and multiple sclerosis [49].

The mechanisms mediating the effects of *HLA* homozygosity on autoimmunity are complex. Even in the case of the extensively studied HLA-B27 and HLA-DQB1, homozygosity does not uniformly increase the risk of disease or alter the clinical phenotype. *HLA-B27* homozygosity in Korean patients with ankylosing spondylitis has no effect on clinical manifestations, functional disability, or radiographic damage [20,21]. *HLA-B27* homozygosity is not associated with increased risk for acute anterior uveitis [50]. Homozygosity for *HLA-DQB1* has no effect on CD clinical outcomes but is associated with anti-tissue transglutaminase antibodies [51]. Therefore, additional *HLA*-related factors are involved in the development of clinical phenotypes including specific allele variants (“suballeles”) and extended haplotypes.

The complex interaction of *HLA* homozygosity with specific alleles and haplotypes is demonstrated in a different CD set of studies where the highest risk for developing CD was conferred by the combination of the *HLA-DQ2.5* heterodimer with HLA-DQB1*02 in homozygosity [52,53,54]. The interaction of homozygosity with extended haplotypes is further complicated by *cis*/*trans* configuration effects in CD [55,56]. Studies in type 1 diabetes examining homozygous *HLA-DR3* (DRB1*03:01-DQA1*05:01-DQB1*02:01) individuals [57], *HLA-DQ8* (HLA-DQA1*03:01-HLA-DQB1*03:02) homozygosity [58], or individual DRB1*03-DQB1*02 and DRB1*04:01-DQB1*03:02 homozygous genotypes [59] also highlight the complexity of the interaction of *HLA* homozygosity with extended haplotypes and the important effects of genotypic epistasis. Finally, the variation in linkage disequilibrium and haplotype blocks of the human MHC loci in a haplotype-specific manner [60] further complicates the interpretation of *HLA* associations with human disease, including the potential effects of homozygosity.

In our study, we introduced and explored the novel concept of LoHLAD which includes *HLA* homozygosity but also encompasses the presence of a single allele or the complete lack of an allele at the *HLA-DRB3/4/5* locus. This unique feature of the *HLA-DRB3/4/5* locus is based on its distinctive linkage disequilibrium with the *HLA-DRB1* locus; specific *HLA-DRB1* alleles (DRB1*01, DRB1*08, DRB1*10) are associated with the lack of an allele at the *HLA-DRB3/4/5* locus [29]. *HLA-DRB3/4/5* genes belong to the *HLA class II* beta chain paralogues that are postulated to have formed as the result of evolutionary duplication of *HLA-DRB1* [61]. In our cohort, the complete lack of an allele at the *HLA-DRB3/4/5* locus was observed exclusively in cases (4 cases with JIA, 2 cases with chronic idiopathic uveitis, and 1 case with SLE); there were no controls with complete lack of an allele at the *HLA-DRB3/4/5* locus. Furthermore, LoHLAD at the *HLA-DRB3/4/5* locus had the strongest association with pediatric-onset autoimmune rheumatic diseases compared to any of the other 4 examined loci (Table 2). This finding may suggest a unique role of the *HLA-DRB3/4/5* locus in pediatric-onset autoimmune rheumatic diseases that is independent of allele specificity.

Our study demonstrates that, across multiple loci, LoHLAD is significantly associated with pediatric-onset autoimmune rheumatic diseases, specifically with JIA, SLE, chronic idiopathic uveitis, and MCTD/UCTD. The lack of association of LoHLAD with LoS, vasculitis, JDM, SS, and SSc relates either to factors inherent to those specific diagnoses or to the small number of such cases in our cohort. The association LoHLAD with pediatric-onset autoimmune rheumatic diseases was observed in all diagnoses with 20 or more cases in our cohort, suggesting that the small number of cases in the remaining diagnoses may account for the lack of association (Table 4). The association of LoHLAD with pediatric-onset autoimmune rheumatic disease was observed in both *HLA-class I* and *HLA-class II* loci but was most notable in class II loci highlighting the prominent role of CD4+ T cells in autoimmunity. Differences between cases and controls were noted at each of the 5 examined loci but were statistically significant only at the *HLA-B*, *HLA-DRB3/4/5*, and *HLA-DQB1* loci. The observed “dose-response effect” (Figure 1C and Table 3) further supports LoHLAD as a positive predictor for pediatric-onset autoimmune rheumatic disease. Remarkably, LoHLAD at 3 or more loci was observed exclusively in cases. Thus, our findings indicate that the higher the number of loci with LoHLAD, the higher the risk for autoimmunity. Additionally, in our study, cases with LoHLAD compared to cases without LoHLAD had significantly earlier symptom onset and shorter time to diagnosis (Supplementary Table S3) suggesting that LoHLAD may be associated with increased phenotypic severity.

Regression models provide additional insight into the significance of LoHLAD as a positive predictor for pediatric-onset autoimmune rheumatic disease. In our study, the comparison of LoHLAD-based models with allele-based models demonstrated that LoHLAD is not inferior to allele specificity as a positive predictor of pediatric-onset autoimmune rheumatic disease. The c-statistics of the best fitted LoHLAD-based model for predicting the presence of either any pediatric-onset autoimmune rheumatic disease or the 4 most common diagnoses in our cohort were higher than the respective c-statistics of the best fitted allele-based model. Most importantly, composite models utilizing both LoHLAD and specific alleles as variables had the best ability in predicting pediatric-onset autoimmune rheumatic disease. Therefore, our data suggest that the combination of LoHLAD and allele specificity provides a novel approach in the assessment of positive autoimmunity risk that may be superior to either LoHLAD-based or allele-based approaches. The results of composite modeling suggest that LoHLAD may have a synergistic effect with allele specificity in increasing the risk for pediatric-onset rheumatic autoimmune disease. The underlying mechanism mediating this potential synergy is unknown. We postulate that high-risk *HLA* alleles present potentially autoreactive peptides and LoHLAD limits the overall diversity of presented peptides thus skewing antigen presentation towards epitopes enriched for autoreactivity.

The complex and multifactorial nature of autoimmune disease immunopathogenesis is evident in our study. LoHLAD was present in a significant percentage of controls (30.60%, Table 2). High-risk alleles such as *HLA-B27*, HLA-DRB1*01, and HLA-DRB1*08 were also present in significant percentages of controls as well (10.4%, 8.2%, and 7.5% respectively; Supplementary Table S7). This observation implies that HLA-related factors are not sufficient to cause autoimmunity and additional factors (genetic, epigenetic, and environmental) are necessary for the development of autoimmune disease.

Our study has strengths as well as limitations. The strengths of our study include the pediatric population, the large sample size, the matching of cases and controls, and the age of cases and controls. The pediatric population may demonstrate the contribution of genetic factors to autoimmunity more clearly than its adult counterpart because, generally, studies in children are less confounded by factors such as environmental exposures and common adult comorbid conditions. The sample size of 413 participants is adequate to provide sufficient data for the study of rare conditions such as pediatric-onset autoimmune rheumatic diseases. Furthermore, cases and controls were evenly matched regarding sex, race, and ethnicity, thus limiting potential bias related to demographic factors. Finally, cases were younger than controls; this difference limits possible bias related to the age of onset of autoimmune rheumatic disease in our cohort.

The limitations of our study include the single center retrospective cohort, the “convenience” control population, and the HLA typing approach. The University of New Mexico pediatric rheumatology clinic is the only clinic in the state of New Mexico providing care to children with rheumatic diseases; therefore, our cohort is at least representative of the pediatric population of the entire state of New Mexico. To provide some form of indirect external validation of the HLA data in our cohort, we performed a secondary analysis examining the association of specific alleles with pediatric-onset autoimmune rheumatic diseases (Supplementary Table S7). This secondary analysis confirmed several known associations such as the associations of JIA with *HLA-A02*, *HLA-B27*, HLA-DRB1*01, HLA-DRB1*08, and HLA-DQB1*04 [62,63,64,65,66,67,68,69,70,71,72,73,74,75]. The greatest limitation of our study is the use of “convenience” controls instead of healthy children. Furthermore, the “convenience” control group in our study may not adequately represent the general pediatric population, introducing potential bias that could affect the generalizability of the results. Nonetheless, the individuals in our control group were formally evaluated by experts from a variety of pediatric subspecialties, and the presence of autoimmune rheumatic disease was ruled out by thorough clinical assessment including, but not limited to, laboratory and imaging studies. The final limitation of our study relates to the *HLA* typing approach that examined only 5 *HLA* loci and was performed by means of low-to-medium resolution sequencing. Although our typing approach provides adequate information to evaluate LoHLAD as a risk factor for autoimmune rheumatic disease, it did not examine all *HLA* loci nor provided potentially important information on the specificity of suballeles or haplotypes, and their contribution to the results of our analysis.

## 5. Conclusions

To conclude, in this proof-of-concept case–control study on autoimmunity risk conferred by the MHC locus, we introduced the novel concept of LoHLAD, utilized LoHLAD as an innovative data analysis framework to investigate *HLA* associations with autoimmunity, and demonstrated that LoHLAD is a significant positive risk factor for the development of pediatric-onset autoimmune rheumatic disease. Analytical approaches utilizing the concept of LoHLAD need to be studied in greater depth, as they can potentially provide new insights into the role of *HLA* genes in autoimmune disease immunopathogenesis, especially when combined with allele specificity. The clinical relevance of LoHLAD as a predictive or diagnostic tool remains to be determined. Our study suggests that the LoHLAD-based approach may be useful in the assessment of autoimmunity risk, but this needs to be externally validated prior to becoming relevant to standard clinical practice. The LoHLAD-based analytical approach is readily applicable in pre-existing *HLA* typing databases, and we encourage clinical researchers to validate our observation on their clinic populations. Future prospective clinical studies will combine data from genetically distinct populations, include healthy individuals as controls, perform typing of all *HLA* loci by means of high-resolution sequencing, and further elucidate the role of LoHLAD in the development of pediatric-onset autoimmune rheumatic disease. Our findings suggest that researchers should consider LoHLAD when analyzing and interpreting the role of *HLA* genes in autoimmunity.

## Figures and Tables

**Table 1:** Study cohort: demographics.

	Controls	Cases	OR [95%CI]	*p*
Age (years), x̄ ± SD (IQR)	13.5 ± 3.8 (4.8)	12.7 ± 3.9 (5.4)	N/A	0.029
Sex, no. (%)			1.61 [0.98, 2.66]	0.069
Female	108/134 (80.6)	201/279 (72)		
Male	26/134 (19.4)	78/279 (28)		
Race, no. (%)			N/A	0.127
White	104/134 (87.4)	199/279 (81.2)		
American Indian or Alaska Native	9/134 (7.6)	34/279 (13.9)		
Black, Asian, Native Hawaiian or	6/134 (5)	12/279 (4.9)		
Other Pacific Islander				
Multiracial or N/D	15/134 (11.2)	34/279 (12.2)		
Ethnicity, no. (%)			0.97 [0.63, 1.49]	0.912
Hispanic	77/127 (60.6)	164/267 (61.4)		
Non-Hispanic	50/127 (39.4)	103/267 (38.6)		
Family history of autoimmunity, no. (%)			2.00 [1.30, 3.10]	0.002
No	57/133 (42.9)	73/268 (27.2)		
Yes	76/133 (57.1)	195/268 (72.8)		

The age distributions of controls and cases were compared by means of Mann–Whitney U test. *p* values and OR were calculated by means of Fisher’s exact test for sex, ethnicity, and positive family history of autoimmunity. The frequency distributions of race between controls and cases were compared by means of χ^2^ test; the OR for race was not calculable as there were more than 2 racial categories in our cohort. The racial categories Black, Asian, Native Hawaiian or Other Pacific Islander were consolidated into a single category to enable statistical comparisons, due to the low numbers of subjects within these categories in our cohort. 15 cases and 34 controls identified as multiracial or preferred not to disclose their race. The missing values for ethnicity (7 controls, 12 cases) and positive family history of autoimmunity (1 control, 11 cases) were excluded from the analysis to enable OR calculations. x̄, mean; SD, standard deviation; IQR, interquartile range; OR, odds ratio; CI, confidence interval; N/D: not disclosed; N/A: not applicable.

**Table 2:** Limitation of *HLA* diversity (LoHLAD) frequency distributions.

LoHLAD	Cases, *n* (%)	Controls, *n* (%)	OR [95%CI]	*p*
Any locus	184 (65.95)	41 (30.60)	4.39 [2.82, 6.84]	<0.0001
*Class I*	54 (19.35)	14 (10.45)	2.06 [1.10, 3.86]	0.031
*Class II*	152 (54.48)	27 (20.15)	4.74 [2.92, 7.69]	<0.0001
*A*	41 (14.70)	14 (10.45)	1.48 [0.78, 2.81]	0.320
*B*	17 (6.09)	1 (0.75)	8.63 [1.14, 65.55]	0.016
*DQB1*	32 (11.47)	5 (3.73)	3.34 [1.27, 8.78]	0.016
*DRB1*	10 (3.58)	2 (1.49)	2.45 [0.53, 11.36]	0.352
*DRB3/4/5*	133 (47.67)	22 (16.42)	4.64 [2.77, 7.75]	<0.0001

LoHLAD frequency distribution comparisons between cases and controls. The analysis examines whether LoHLAD was present at any of the 5 examined *HLA* loci (“Any locus”), any of the two *HLA-class I* loci (“Class I”), any of the three *HLA-class II* loci (“Class II”), or each separate *HLA* locus (*A*, *B*, *DQB1*, *DRB1*, *DRB3/4/5*). OR were calculated by means of Fisher’s exact test. *p* values have been adjusted for multiple comparisons. LoHLAD, Limitation of *HLA* Diversity; OR, odds ratio; CI, confidence interval.

**Table 3:** Limitation of *HLA* diversity (LoHLAD) cumulative effect.

Number of Loci	Cases, *n* (%)	Controls, *n* (%)	OR [95%CI]	*p*
0	95 (34.05)	93 (69.40)	N/A	N/A
1	151 (54.12)	38 (28.36)	3.89 [2.47, 6.14]	<0.0001
2	20 (7.17)	3 (2.24)	6.53 [1.88, 22.70]	0.0007
3	10 (3.58)	0 (0)	N/A	N/A
4	2 (0.72)	0 (0)	N/A	N/A
5	1 (0.36)	0 (0)	N/A	N/A
≥2	33 (11.83)	3 (2.24)	10.77 [3.19, 36.33]	<0.0001

The cumulative effect of LoHLAD (“dose–response effect”) was examined by comparing the frequencies of the number of loci with LoHLAD between cases (*n* = 279) and controls (n = 134). OR and *p* values were calculated by means of Fisher’s exact test. Cases and controls with no LoHLAD (“0”) were used as the reference point for all comparisons. Because there were no controls with more than 2 loci with LoHLAD, no OR could be calculated for categories “3”, “4”, and “5” (first column). Therefore, a new category was created signifying the presence of LoHLAD in 2 or more loci (“≥2”, last row) in order to perform a valid comparison that includes cases and controls with “3”, “4”, and “5” in the first column. LoHLAD, Limitation of *HLA* Diversity; OR, odds ratio; CI, confidence interval; N/A, not applicable.

**Table 4:** Diagnosis-specific limitation of *HLA* diversity (LoHLAD) risk assessment.

LoHLAD, *n* (%)		OR [95%CI]	*p*
Controls (*n* = 134)	41 (30.6)	N/A	N/A
JIA (*n* = 134)	98 (73.1)	6.18 [3.63, 10.49]	<0.0001
SLE (*n* = 43)	26 (60.5)	3.47 [1.70, 7.08]	0.002
Chronic idiopathic uveitis (*n* = 31)	23 (74.2)	6.52 [2.69, 15.79]	<0.0001
MCTD / UCTD (*n* = 20)	14 (70)	5.29 [1.90, 14.74]	0.007
LoS (*n* = 16)	7 (43.8)	1.76 [0.62, 5.06]	0.664
Vasculitis (*n* = 14)	7 (50)	2.27 [0.75, 6.88]	0.362
JDM (*n* = 12)	3 (25)	0.76 [0.20, 2.94]	1.000
SS (*n* = 6)	4 (66.7)	4.54 [0.80, 25.76]	0.280
SSc (*n* = 3)	2 (66.7)	4.54 [0.40, 51.45]	0.618

OR and *p* values for the presence of LoHLAD at any locus between controls and diagnosis-specific groups cases were calculated by means of Fisher’s exact test. *p* values have been adjusted for multiple comparisons. LoHLAD, Limitation of *HLA* Diversity; OR, odds ratio; CI, confidence interval; N/A, not applicable; JIA, Juvenile Idiopathic Arthritis; SLE, Systemic Lupus Erythematosus; MCTD, Mixed Connective Tissue Disease; UCTD, Undifferentiated Connective Tissue Disease; LoS, Localized Scleroderma; JDM, Juvenile Dermatomyositis; SS, Sjögren’s Syndrome; SSc, Systemic Sclerosis; N/A: not applicable.

## Data Availability

De-identified data are available upon reasonable request with a transfer agreement.

## Supplementary Materials

The following supporting information can be downloaded at: https://www.mdpi.com/article/10.3390/jcm14030916/s1, Figure S1: Overview of study design.; Table S1: Study cohort (cases): diagnostic categories; Table S2: Diagnosis-specific limitation of *HLA* diversity (LoHLAD) cumulative effect; Table S3: Limitation of HLA diversity (LoHLAD), age at symptom onset and time to diagnosis; Table S4: Correlations between logistic regression model variables; Table S5: c-statistic values for logistic regression models; Table S6: Detailed model evaluation; Table S7: Positive risk assessment of HLA alleles. References [22,23,24,25,26,27,28] are cited in the supplementary materials
