# Supplementary material for: The Limitation of HLA Diversity as a Risk Factor for Pediatric-Onset Autoimmune Rheumatic Disease
Source: J Clin Med. 2025 Jan 30;14(3):916. doi: 10.3390/jcm14030916 (PMC11818087; doi:10.3390/jcm14030916)
Supplement: Supplementary file 1 [file jcm-14-00916-s001.zip › jcm-3355070-supplementary.pdf]

SUPPLEMENTARY MATERIAL

The Limitation of HLA Diversity as a Risk Factor for Pediatric-Onset Autoimmune Rheumatic Disease

Ioannis Kalampokis [1,2,*], Craig S. Wong [2], Jihyun Ma [1], Lynette M. Smith [1], Barbara J. Masten [2,3], Devon Chabot-Richards [2] and David S. Pisetsky [4,5]

[1] University of Nebraska Medical Center, Omaha, NE 68198, USA; jihyun.ma@unmc.edu (J.M.); lmsmith@unmc.edu (L.M.S.)
[2] University of New Mexico, Albuquerque, NM 87106, USA; cwong@salud.unm.edu (C.S.W.); bmasten@salud.unm.edu (B.J.M.); dchabot-richards@salud.unm.edu (D.C.-R.)
[3] Tricore Reference Laboratories, Albuquerque, NM 87102, USA
[4] Duke University Medical Center, Durham, NC 27710, USA; david.pisetsky@duke.edu
[5.] Durham Veterans Administration Medical Center, Durham, NC 27705, USA
* Correspondence: ikalampokis@unmc.edu or ioannis.kalampokis@gmail.com; Tel.: +1-(917)-975-9513

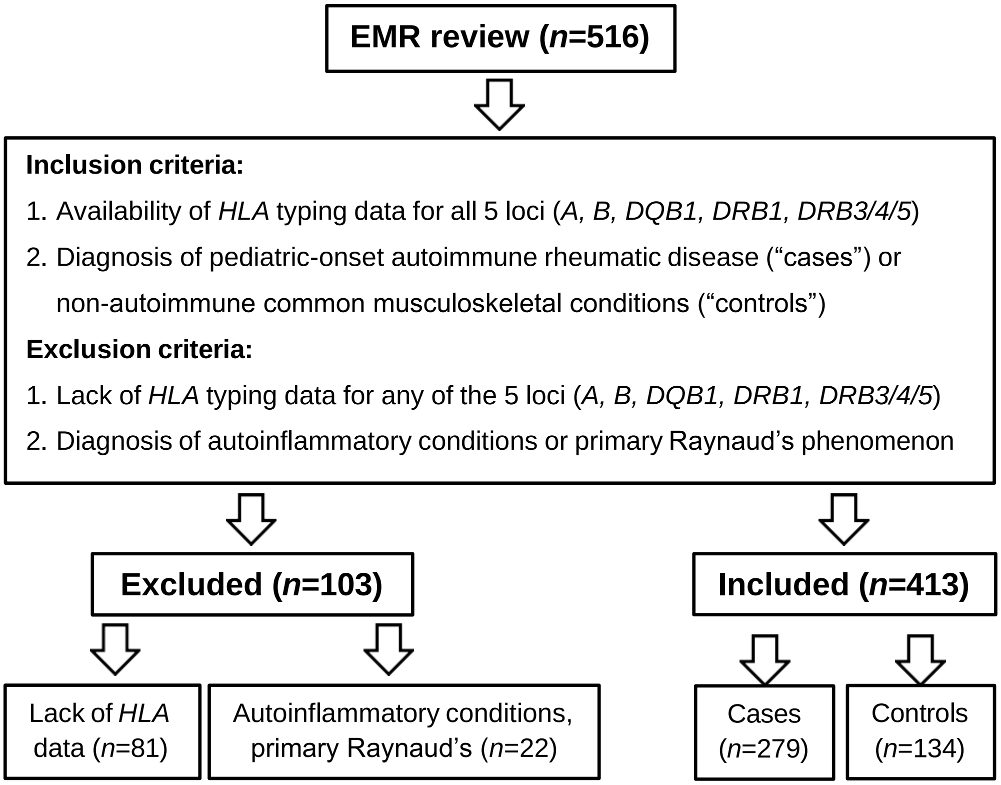

Figure S1. Overview of study design. We conducted a proof-of- concept case-control observational study examining the limitation of HLA diversity as an allele-independent risk factor for pediatric-onset autoimmune rheumatic disease. We reviewed the electronic medical records (EMR) of 516 unique individuals. The inclusion criteria were satisfied by 413 subjects, 279 with pediatric-onset autoimmune rheumatic disease (cases) and 134 with non-autoimmune common musculoskeletal conditions (controls). 103 subjects were excluded from the study, 81 due to the lack of HLA typing data and 22 due to their diagnosis of autoinflammatory conditions or primary Raynaud's phenomenon.

Table S1. Study cohort (cases): diagnostic categories

Diagnosis	*n* (%)[A]	*n* (%)[B]
JIA	16 (5.7)	
Oligoarticular		41 (30.6)
Polyarticular		50 (37.3)
ERA		33 (24.6)
Systemic-onset		8 (6)
Psoriatic		2 (1.5)
SLE	43 (15.4)	
Chronic idiopathic uveitis	31 (11.1)	
MCTD / UCTD	20 (7.2)	
MCTD		12 (60)
UCTD		8 (40)
LoS	16 (5.7)	
Vasculitis	14 (5)	
ANCA positive		8 (57.1)
ANCA negative		3 (21.4)
IgA vasculitis		2 (14.3)
Behçet's disease		1 (7.1)
JDM	12 (4.3)	
SS	6 (2.2)	
SSc	3 (1.1)	
Diffuse cutaneous		2 (66.7)
Limited cutaneous	134 (48)	1 (33.3)

[A]Specific pediatric-onset autoimmune rheumatic disease diagnoses as absolute numbers and percentages of all cases. [B]For certain diagnoses (JIA, MCTD/UCTD, vasculitis, SSc), the absolute numbers and percentages of disease subtypes within a diagnostic category are also presented. JIA, Juvenile Idiopathic Arthritis; ERA, Enthesitis-Related Arthritis; SLE, Systemic Lupus Erythematosus; MCTD, Mixed Connective Tissue Disease; UCTD, Undifferentiated Connective Tissue Disease; LoS, Localized Scleroderma; ANCA, Anti-Neutrophil Cytoplasmic Antibodies; JDM, Juvenile Dermatomyositis; SS, Sjögren's Syndrome; SSc, Systemic Sclerosis.

Table S2. Diagnosis-specific limitation of *HLA* diversity (LoHLAD) cumulative effect

	HLA loci with LoHLAD, *n* (% within category)						
	0	1	2	3	4	5	*P*
Controls (*n*=134)	93 (69.40)	38 (28.36)	3 (2.24)	0 (0.00)	0 (0.00)	0 (0.00)	N/A
JIA (*n*=134)	36 (26.87)	81 (60.45)	11 (8.21)	4 (2.99)	1 (0.75)	1 (0.75)	*<0.0001*
SLE (*n*=43)	17 (39.53)	21 (48.84)	3 (6.98)	1 (2.33)	1 (2.33)	0 (0.00)	*<0.0001*
Chronic idiopathic uveitis (*n*=31)	8 (25.81)	21 (67.74)	2 (6.45)	0 (0.00)	0 (0.00)	0 (0.00)	*<0.0001*
MCTD / UCTD (*n*=20)	6 (30.00)	10 (50.00)	2 (10.00)	2 (10.00)	0 (0.00)	0 (0.00)	*<0.0001*
LoS (*n*=16)	9 (56.25)	5 (31.25)	2 (12.50)	0 (0.00)	0 (0.00)	0 (0.00)	0.141
Vasculitis (*n*=14)	7 (50.00)	6 (42.86)	0 (0.00)	1 (7.14)	0 (0.00)	0 (0.00)	0.073
JDM (*n*=12)	9 (75.00)	2 (16.67)	0 (0.00)	1 (8.33)	0 (0.00)	0 (0.00)	0.786
SS (*n*=6)	2 (33.33)	3 (50.00)	0 (0.00)	1 (16.67)	0 (0.00)	0 (0.00)	*0.015*
SSc (*n*=3)	1 (33.33)	2 (66.67)	0 (0.00)	0 (0.00)	0 (0.00)	0 (0.00)	0.583

The cumulative effect of LoHLAD ("dose-response effect") was examined by comparing the frequency distributions of the number of loci with LoHLAD between diagnosis-specific case groups and controls. Controls were used as the reference for all comparisons. *P* values were calculated by means of the Cochran-Armitage trend test. N/A, not applicable; JIA, Juvenile Idiopathic Arthritis; SLE, Systemic Lupus Erythematosus; MCTD, Mixed Connective Tissue Disease; UCTD, Undifferentiated Connective Tissue Disease; LoS, Localized Scleroderma; JDM, Juvenile Dermatomyositis; SS, Sjögren's Syndrome; SSc, Systemic Sclerosis.

Table S3. Limitation of *HLA* diversity (LoHLAD), age at symptom onset and time to diagnosis

	Age at symptom onset		Time to diagnosis	
	LoHLAD-	LoHLAD+	LoHLAD-	LoHLAD+
Mean, years	8.90	7.46	1.31	0.90
SD, years	4.35	4.38	2.20	1.64
SEM, years	0.45	0.32	0.23	0.12
Median, years	9.50	7.50	0.50	0.50
IQR, years	7.50	6.88	1.50	1.00
P	*0.008*		*0.021*	

Comparisons of the age at symptom onset and time to diagnosis of pediatric- onset autoimmune rheumatic disease in cases in relation to the presence ("LoHLAD+") or absence ("LoHLAD-") of limitation of *HLA* diversity at any of the 5 examined loci. *P* values were calculated by means of Mann- Whitney U test. SD, standard deviation; SEM, standard error of mean; IQR, interquartile range.

Table S4. Correlations between logistic regression model variables

	LoHLAD					Allele					
	A	*B*	*DQB1*	*DRB1*	*DRB3/4/5*	*A02*	*A24*	*B27*	*DRB1*01*	*DRB1*08*	*DQB1*04*
**LoHLAD**											
A	1	0.09	-0.01	0.03	-0.1	0.12	-0.11	0	-0.05	-0.08	-0.1
B	0.09	1	0.09	0.14	0.05	0.05	-0.01	0.11	-0.01	-0.03	0
DQB1	-0.01	0.09	1	***0.42***	-0.01	0.11	0.14	0	-0.03	-0.16	-0.17
DRB1	0.03	0.14	***0.42***	1	0.14	0.1	0.02	-0.03	-0.07	-0.08	-0.09
DRB3/4/5	-0.1	0.05	-0.01	0.14	1	0.1	0.06	-0.01	***0.56***	***0.63***	***0.51***
**Allele**											
A02	0.12	0.05	0.11	0.1	0.1	1	-0.14	-0.04	-0.06	0.14	0.14
A24	-0.11	-0.01	0.14	0.02	0.06	-0.14	1	0.14	-0.04	0.12	0.1
B27	0	0.11	0	-0.03	-0.01	-0.04	0.14	1	-0.01	0.01	0.05
*DRB1*01*	-0.05	-0.01	-0.03	-0.07	***0.56***	-0.06	-0.04	-0.01	1	-0.04	-0.09
*DRB1*08*	-0.08	-0.03	-0.16	-0.08	***0.63***	0.14	0.12	0.01	-0.04	1	***0.84***
*DQB1*04*	-0.1	0	-0.17	-0.09	***0.51***	0.14	0.1	0.05	-0.09	***0.84***	1

The Pearson correlation coefficients of all variables used in multivariate logistic regression models are presented. Statistically significant correlations are highlighted in bold/italics (P <0.05). LoHLAD, limitation of *HLA* diversity.

Table S5. c-statistic values for logistic regression models

	Controls (*n*=134) vs.					
	All cases (*n*=279)	Diagnoses with *n*≥20 (*n*=228)	JIA (*n*=134)	SLE (*n*=43)	Uveitis (*n*=31)	MCTD/UCTD (*n*=20)
**LoHLAD-based models**						
Number of "hits" (0-5)	0.688	0.711	0.724	0.661	0.720	0.721
Any locus	0.677	0.700	0.713	0.649	0.718	0.697
Class I	0.545	0.549	0.556	0.541	0.504	0.598
Class II	0.672	0.693	0.694	0.655	0.754	0.674
Class I, Class II	0.702	0.727	0.737	0.676	0.758	0.726
A	0.521	0.527	0.534	0.518	0.504	0.548
B	0.527	0.525	0.526	0.531	0.504	0.546
DQB1	0.539	0.541	0.545	0.551	0.503	0.556
DRB1	0.510	0.510	0.511	0.504	0.507	0.543
DRB3/4/5	0.656	0.677	0.679	0.627	0.757	0.643
A, B, DRB1, DRB3/4/5	0.689	0.713	0.727	0.642	0.767	0.721
A, B, DQB1, DRB3/4/5	***0.711***	***0.735***	***0.749***	***0.677***	***0.769***	***0.725***
**Allele-based models**						
A02	0.550	0.558	0.586	0.571	0.543	0.505
A24	0.550	0.549	0.567	0.503	0.588	0.540
B27	0.534	0.544	0.578	0.517	0.528	0.527
*DRB1*01*	0.554	0.601	0.571	0.529	0.604	0.534
*DRB1*08*	0.590	0.564	0.601	0.591	0.640	0.563
*DQB1*04*	0.566	0.577	0.582	0.576	0.614	0.513
*A02, A24, B27, DRB1*01, DQB1*04*	0.672	0.694	0.735	0.65	0.742	0.563
*A02, A24, B27, DRB1*01, DRB1*08*	***0.698***	***0.713***	***0.753***	***0.669***	***0.780***	***0.627***
**Composite models**						
*A, DRB1, A02, A24, B27, DRB1*01, DQB1*04*	0.694	0.716	0.760	0.660	0.755	0.629
*A, B, DRB1, A02, A24, B27, DRB1*01, DRB1*08*	0.716	0.738	0.777	0.678	0.777	0.715
*A, B, DQB1, A02, A24, B27, DRB1*01, DQB1*04*	0.706	0.730	0.768	0.699	0.752	0.669
*A, B, DQB1, A02, A24, B27, DRB1*01, DRB1*08*	0.732	0.753	0.787	0.708	0.774	0.737
A, B, DQB1, DRB3/4/5, A02, A24, B27	***0.740***	***0.764***	***0.796***	***0.704***	***0.822***	***0.739***

Column titles represent the predicted outcome and row titles represent the variable(s) used as predictors. All outcomes are binary. The "Number of hits" predictor represents the number of loci with LoHLAD and is used as an ordinal variable in a univariate model; all other models, both univariate and multivariate, use binary variables. The "Any locus" predictor designates LoHLAD at any of the 5 examined loci and is used as a binary variable in a univariate model. The rest of LoHLAD-based univariate models used LoHLAD in either *class I, class II, A, B, DQB1, DRB1, or DRB3/4/5.* Three LoHLAD-based multivariate models with binary variables are shown (*"Class I, Class II", "A, B, DRB1, DQB1", and "A, B, DQB1, DRB3/4/5"*). Allele-based models utilized 6 specific alleles (*A02, A24, B27, DRB1*01, DRB1*08, DQB1*04*). We constructed 6 allele-based univariate models and 2 multivariate allele-based models. Composite models utilized both LoHLAD-based and allele-based variables. JIA: Juvenile Idiopathic Arthritis, SLE: Systemic Lupus Erythematosus, MCTD: Mixed Connective Tissue Disease, UCTD: Undifferentiated Connective Tissue Disease, LoHLAD: Limitation of *HLA* Diversity.

Table S6. Detailed model evaluation

Testing global null hypothesis: beta=0

**Test**	**DF**	χ^2	***P***
Likelihood Ratio	4	68.9338	<0.0001
Score	4	58.9124	<0.0001
Wald	4	53.1577	<0.0001

Analysis of maximum likelihood estimates

**Parameter**	**DF**	**Estimate**	**SE**	**Wald** χ^2	***P***
Intercept	1	-0.3401	0.1580	4.6360	0.0313
A	1	0.8637	0.3621	5.6890	0.0171
B	1	2.1543	1.0763	4.0065	0.0453
DQB1	1	1.5145	0.5249	8.3257	0.0039
DRB3/4/5	1	1.8535	0.2783	44.3672	<0.0001

Odds ratio estimates

**Effect**	**Point Estimate**	**95% Wald CI**
A	2.372	1.166, 4.823
B	8.622	1.046, 71.073
DQB1	4.547	1.625, 12.722
DRB3/4/5	6.382	3.699, 11.011

Model fit statistics

**Criterion**	**Intercept Only**	**Intercept and Covariates**
AIC	479.148	418.214
SC	483.039	437.672
-2 Log L	477.148	408.214

Association of predicted probabilities and observed responses

**% Concordant**	59.3	**Somers' D**	0.469
**% Discordant**	12.4	**Gamma**	0.654
**% Tied**	28.3	**Tau-a**	0.219
**Pairs**	30552	**c-statistic**	0.735

Detailed evaluation parameters of the best fitted LoHLAD- based multivariate logistic regression model in predicting the presence of the 4 most common autoimmune disease diagnoses in our cohort (JIA, SLE, idiopathic uveitis, MCTD/UCTD). χ^2, chi square; SE, standard error; LoHLAD, Limitation of *HLA* Diversity; DF, Degrees of Freedom; AIC, Akaike Information Criterion; SC, Schwarz Criterion; JIA, Juvenile Idiopathic Arthritis; SLE, Systemic Lupus Erythematosus; MCTD, Mixed Connective Tissue Disease; UCTD, Undifferentiated Connective Tissue Disease.

Table S7. Positive risk assessment of *HLA* alleles

Diagnosis	Allele	Cases, *n* (%)	Controls *n* (%)	OR [95% CI	*P*
Any (*n*=279)	*DRB1*01*	53 (19)	11 (8.2)	2.62 [1.32, 5.20]	*0.012*
	*DRB1*08*	71 (25.4)	10 (7.5)	4.23 [2.11, 8.51]	*<0.0001*
	*DQB1*04*	72 (25.8)	17 (12.7)	2.40 [1.35, 4.26]	*0.005*
JIA (*n*=134)	*A02*	82 (61.2)	59 (44)	2.00 [1.23, 3.26]	*0.031*
	A24	42 (31.3)	24 (17.9)	2.09 [1.18, 3.71]	*0.031*
	B27	35 (26.1)	14 (10.4)	3.03 [1.54, 5.95]	*0.007*
	*DRB1*01*	30 (22.4)	11 (8.2)	3.23 [1.54, 6.75]	*0.005*
	*DRB1*08*	37 (27.6)	10 (7.5)	4.73 [2.24, 9.99]	*0.0001*
	*DQB1*04*	39 (29.1)	17 (12.7)	2.83 [1.50, 5.31]	*0.004*
SLE (*n*=43)	*DRB1*08*	11 (25.6)	10 (7.5)	4.26 [1.66, 10.92]	0.033
Uveitis (*n*=31)	*DRB1*01*	9 (29)	11 (8.2)	4.57 [1.70, 12.32]	*0.013*
	*DRB1*08*	11 (35.5)	10 (7.5)	6.82 [2.56, 18.13]	*0.001*
	*DQB1*04*	11 (35.5)	17 (12.7)	3.79 [1.55, 9.26]	*0.015*

Specific HLA alleles significantly associated (*P*<0.05) with increased (positive) risk for pediatric-onset autoimmune rheumatic disease in study cohort are presented. Risk assessments are reported for any diagnosis ("Any"), and for the 3 most common diagnoses in our cohort (JIA, SLE, chronic idiopathic uveitis). Odds ratios (OR) and *P* values were calculated by means of Fisher's exact test. *P* values have been adjusted for multiple comparisons by means of false discovery rate (the adjustment included only alleles with *P*<0.05). No alleles were significantly associated with increased risk for MCTD/UCTD, LoS, vasculitis, JDM, SS, or SSc. OR, odds ratio; CI, confidence interval; JIA, Juvenile Idiopathic Arthritis; SLE, Systemic Lupus Erythematosus; MCTD, Mixed Connective Tissue Disease; UCTD, Undifferentiated Connective Tissue Disease; LoS, Localized Scleroderma; JDM, Juvenile Dermatomyositis; SS, Sjögren's Syndrome; SSc, Systemic Sclerosis.
